# Properties of Metallic and Oxide Thin Films Based on Ti and Co Prepared by Magnetron Sputtering from Sintered Targets with Different Co-Content

**DOI:** 10.3390/ma14143797

**Published:** 2021-07-07

**Authors:** Damian Wojcieszak, Michał Mazur, Patrycja Pokora, Adriana Wrona, Katarzyna Bilewska, Wojciech Kijaszek, Tomasz Kotwica, Witold Posadowski, Jarosław Domaradzki

**Affiliations:** 1 Faculty of Microsystem Electronics and Photonics, Wroclaw University of Science and Technology, Janiszewskiego 11/17, 50-372 Wroclaw, Poland; damian.wojcieszak@pwr.edu.pl (D.W.); michal.mazur@pwr.edu.pl (M.M.); wojciech.kijaszek@pwr.edu.pl (W.K.); tomasz.kotwica@pwr.edu.pl (T.K.); witold.posadowski@pwr.edu.pl (W.P.); jaroslaw.domaradzki@pwr.edu.pl (J.D.); 2 Lukasiewicz Research Network—Institute of Non-Ferrous Metals, Gliwice Division, Sowinskiego 5, 44-100 Gliwice, Poland; adriana.wrona@imn.lukasiewicz.gov.pl (A.W.); katarzyna.bilewska@imn.lukasiewicz.gov.pl (K.B.)

**Keywords:** amorphous thin film, magnetron sputtering, titanium, cobalt, memristive-like effect, transparent electronics

## Abstract

In this work, selected properties of metallic and oxide thin films based on titanium and cobalt were described. Thin-film coatings were prepared using the magnetron sputtering method. The deposition was carried out from sintered targets with different Co-content (2 at.%, 12 at.% and 50 at.%). The relation between the Ti–Co target composition and the Co-content in the metallic and oxide films was examined. There was 15–20% more cobalt in the films than in the target. Moreover, the deposition rate under neutral conditions (in Ar plasma) was even 10-times higher compared to oxidizing Ar:O_2_ (70:30) plasma. A comprehensive analysis of the structural properties (performed with GIXRD and SEM) revealed the amorphous nature of (Ti,Co)Ox coatings, regardless of the cobalt content in the coating. The fine-grained, homogenous microstructure was observed, where cracks and voids were identified only for films with high Co-content. Optical studies have shown that these films were well transparent (60% ÷ 80%), and the amount of cobalt in the target from which they were sputtered had a significant impact on the decrease in the transparency level, the slight shift of the absorption edge position (from 279 nm to 289 nm) as well as the decrease in their optical band gap energy (from 3.13 eV to 1.71 eV). Electrical studies have shown that in (Ti,Co)Ox thin films, a unipolar memristive-like effect can be observed. The occurrence of such effects has not been reported so far in the case of TiO_2_ coatings with the addition of Co.

## 1. Introduction

The progress in electronic components functionality is related to the development of, among others, a new type of multifunctional thin-film coatings that will combine the features and attributes of various materials. Among the materials currently used in optoelectronics, titanium dioxide is well recognized whose production technology is mastered and has a wide application area. It is a very good matrix due to high chemical, thermal and mechanical resistance. This wide band gap oxide (ca. 3 eV) is neutral to the environment and exhibits high transparency for light in a wide spectral range (320 nm–6000 nm), has a high refractive index (2.4–2.7), and exhibits high photocatalytic activity [1,2]. TiO_2_ is used in the construction of optical filters [3], in solar cells [4] and as an active layer in gas sensors [5]. The properties of such transition metal oxides as TiO_2_ can be modified by a proper selection of deposition parameters, post-process treatment (e.g., annealing) or doping. Many additives and admixtures are used to modify the properties of TiO_2_. Cobalt can also be included, but its influence on the properties of titanium dioxide is not yet well understood. There are no comprehensive studies on thin films based on titanium and cobalt (especially in the form of oxides) that limit the possibility of fully exploiting the potential that the combination of these two materials can offer. Cobalt and its oxides exhibit high stability (especially in the form of CoO and Co_3_O_4_) [6], as well as high photocatalytic activity [7] or ferromagnetic properties [8]. Oxides and metallic materials based on Co can be applied in spintronics [9], magneto-optical devices [10], in the construction of semiconductor sensors [11], electrochromic coatings [12] and heterogeneous catalysts [13]. Materials based on titanium and cobalt constitute a “new group” of materials whose properties have not been fully understood and used so far. Doping titanium dioxide with cobalt allows to modify a number of its properties, primarily: structural [14,15,16,17,18,19,20,21], optical [14,15,16,17,18,20,21], electrical [15,16,20,21] and magnetic [20]. These coatings have been of great interest due to the formation of dilute magnetic semiconductors (DMS), which is promising for the manufacturing of spintronic and magneto-optic devices [22,23,24]. Co-doped TiO_2_ is a ferromagnetic material at room temperature [25] that turns it into a promising material for magneto-resistive random-access memory (MRAM) [24].

The properties of doped coatings based on titanium and its oxides largely depend on the method of their preparation. Among the many methods and their use in the electronics industry, attention should be paid to magnetron sputtering. It requires the use of targets that are the source of the sputtered material over which the plasma is ignited. Unfortunately, due to the magnetic properties of cobalt, it closes the magnetic field, and it is difficult to ignite the plasma over a magnetron with such a target. A solution can be the use of thin Co targets or multicomponent targets such as Ti–Co. This paper presents studies of the structural, optical and electrical properties of thin (Ti,Co)Ox coatings. They were obtained by magnetron sputtering, and the analysis of their properties was described from the stage of sintering Ti–Co targets (with different material compositions) using the SPS method. It was necessary to determine the relationship between the cobalt content in the target and its final amount in both the metallic and oxide films. The results of electrical measurements have shown that in (Ti,Co)Ox thin films, a unipolar memristive-like effect can be observed. The occurrence of such effects has not been reported so far in the case of TiO_2_ coatings with the addition of Co. This means that materials based on titanium and cobalt oxides may be prospective for electronics, not only due to their well-known spintronic properties but also because of their memristive-like behavior.

## 2. Experimental

### 2.1. Preparation of Targets and Thin Films

Metallic and oxide thin films were prepared using the magnetron sputtering method. For the preparation, the Ti–Co targets with 2 at.%, 12 at.% and 50 at.% of Co-content were used. The targets were prepared by spark plasma sintering method (SPS) with the aid of the system provided by the FCT GmbH company [26,27]. Targets were sintered at 1200 °C in a graphite matrix. They had a diameter of 30 mm and a thickness of 3 mm. The material composition of the targets was determined by a JXA-8230 X-ray microanalyzer (JEOL) with wave and energy dispersion spectrometers (WDS and EDS). These targets were used for the preparation of metallic and oxide thin films (Ti–Co and (Ti,Co)Ox, respectively). Pre-cleaning of the surface of targets and substrates was applied (low-pressure plasma discharge in the chamber: AC supply, 170 W, 1.2 × 10^−1^ mbar, 3 min). Deposition processes were carried out using either pure Ar or a mixture of Ar:O_2_ (70:30) as a working and reactive gas. The constant gas flow was set during the entire deposition process at 26 sccm that was controlled by the ERG1MPSc unit (BetaERG). Films, as deposited in the argon atmosphere, were metallic, while the addition of 30% of oxygen was sufficient to obtain the full oxidation of the coatings. The pressure in a vacuum chamber during the process was 1.5 × 10^−2^ mbar. The magnetron was powered by an MSS2 (2 kW) pulsed DC power supply unit (DORA Power System). The deposition process lasted 60 min for metallic and 120 min for oxide thin films. The substrates were placed on a rotating drum at a 100 mm distance from the sputtered target. Sputtering rate (SR) was calculated taking into consideration the film thickness and the time of material deposition on the given substrate holder area, which is sputtering time divided into 6. Films were deposited on fused silica (SiO_2_) and silicon wafers.

### 2.2. Methods of Targets and Thin Film Characterization

The thickness and surface topography of as-deposited thin films was determined by the optical profiler Talysurf CCI (Taylor Hobson, Leicester, UK). Based on three-dimensional profiles, the analysis of surface roughness was performed, and the value of root mean square roughness (Sq) was determined.

Surface observation and material composition of the metallic coatings was determined by a JXA-8230 (JEOL) X-ray microanalyzer equipped with WDS and EDS spectrometers. In the case of oxide (Ti,Co)Ox films, the observations of surface and cross-section morphology were performed with the use of FEI Helios NanoLab 600i SEM coupled with an EDS spectrometer. Comparison of the intensity of CoKα and TiKα emission lines allowed to determine the amount of each element.

The structure of the oxide coatings was determined using X-ray diffraction in grazing incidence mode (GIXRD) with the aid of Empyrean PIXel3D (Panalytical, Malvern, UK) diffractometer with Cu Kα radiation (0.15406 nm). The X-ray source was powered with 40 kV and 30 mA. The diffraction patterns were collected with the step size equal to 0.05° in the 2theta range of 20°–80° and time per step equal to 2 s. The incidence angle was constant and equal to 3° to the sample surface. For data analysis, MDI JADE 5.0 software (ICDD, Newtown Square, PA, USA) was used.

The optical properties of oxide coatings were analyzed based on transmission characteristics. Measurements were conducted in the wavelength range of 250–900 nm using Ocean Optics QE65000 spectrophotometer (Ocean Optics, Largo, FL, USA) and a coupled deuterium-halogen light source. The analysis carried out based on the obtained data allowed to determine the cut-off wavelength (λ_cut-off_) and optical band gap energy (E_g_^opt^).

Electrical properties were determined based on the DC current-to-voltage electrical measurements using a Keithley SCS4200 semiconductor characterization system (Keithley Instruments LLC, Cleveland, OH, USA) and an M100 probe station (Cascade Microtech, Beaverton, OR, USA). The setup for the measurement of the capacitance–voltage characteristics was also additionally equipped with an Agilent 4294A precision impedance analyzer (Agilent Technologies Inc., Santa Clara, CA, USA) To allow electrical tests, circular gold 1 mm pads were evaporated at the top of the (Ti,Co)Ox thin film deposited on the silicon substrate. Measurements of C–V characteristics were performed for 100 equally repeated cycles.

## 3. Results and Discussion

Thin-film coatings based on Ti and Co can have a wide application area in electronic and optoelectronic devices. As mentioned, especially transparent oxide coatings based on Ti–Co oxides can be used in many different areas. However, defining their properties (currently very poorly understood and described in the literature) requires reproducible mass-scale manufacturing, which can be provided by, e.g., magnetron sputtering. In this paper, first, the influence of the working gas atmosphere (neutral—Ar and oxidizing Ar:O_2_) in which Ti–Co targets were sputtered on the properties of the prepared coatings is discussed. The effect of Co-content in the target on the deposition rate was investigated. The next part is devoted to the analysis of a very broad characterization of the structural, optical and electrical properties of oxide (Ti,Co)Ox coatings in relation to their potential areas of application in electronics and optoelectronics.

### 3.1. Influence of Ti–Co Target Composition on Its Sputtering Conditions

#### 3.1.1. Material Composition of Sintered Ti–Co Targets

**Figure 1:**
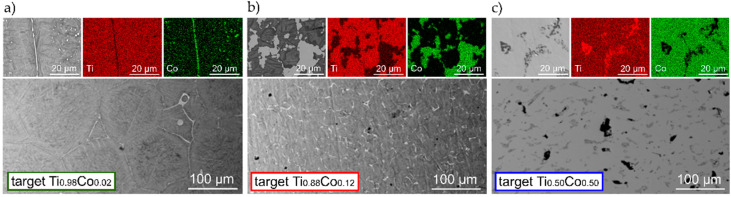
Microstructure with WDS maps of elemental distribution of Ti and Co in sintered targets: (**a**) Ti_0.98_Co_0.02_, (**b**) Ti_0.88_Co_0.12_, (**c**) Ti_0.50_Co_0.50_.

In Figure 1, the microstructure with a WDS-map of the elemental distribution of the targets is shown. It was found that the Co-content in the three prepared targets was ca. 2 at.%, 12 at.% and 50 at.% (the exact numbers are collected in Table 1). The distribution of the elements in the Ti_0.98_Co_0.02_ target indicates that cobalt is dissolved in titanium in the entire volume of the target (Figure 1a). Cobalt content in the Ti_0.98_Co_0.02_ target is slightly different from the assumed one (1.7 at.%). This may be related due to the accuracy of the method or the effect of material segregation in the volume of the entire target. In the case of the Ti_0.88_Co_0.12_ target, high segregation of elements can be macroscopically observed. In the center of the Ti_0.88_Co_0.12_ target, the presence of a bright area of the Ti–Co phase and black ones of titanium are observed—most probably due to low cobalt dissolution (Figure 1b). The results of the WDS analysis of the Ti_0.50_Co_0.50_ target suggest a finely divided structure with visible dark areas which are richer in titanium (Figure 1c).

#### 3.1.2. Material Composition of Metallic Ti–Co Thin Films

**Figure 2:**
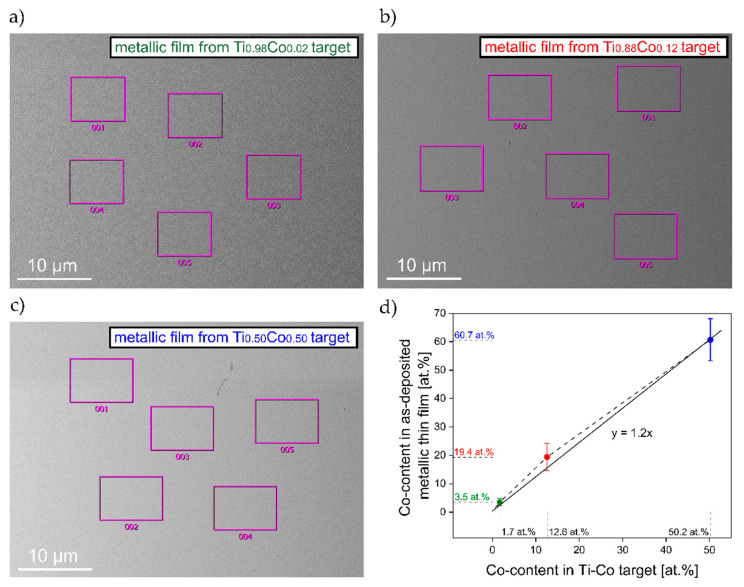
Images of surface topography with marked areas of EDS analysis for metallic Ti–Co thin films as deposited from: (**a**) Ti_0.98_Co_0.02_, (**b**) Ti_0.88_Co_0.12_, (**c**) Ti_0.50_Co_0.50_ targets; with (**d**) a diagram of the dependence between Co-content in the target and in the metallic film.

In Figure 2, images of surface topography with marked areas of EDS analysis for metallic Ti–Co thin films as deposited from Ti_0.98_Co_0.02_, Ti_0.88_Co_0.12_ and Ti_0.50_Co_0.50_ targets are shown.

Based on these results, a diagram of the relation between Co-content in the target and in the metallic film is shown in Figure 2d. The EDS results (obtained for five randomly selected areas on each sample) showed that the distribution of cobalt in the metallic films was homogeneous. It was also found that the Co-amount in the films was relatively 20% higher than in the targets. Detailed results of the material composition research of metallic coatings are summarized in Table 1.

#### 3.1.3. Material Composition of Oxide (Ti,Co)Ox Thin Films

**Figure 3:**
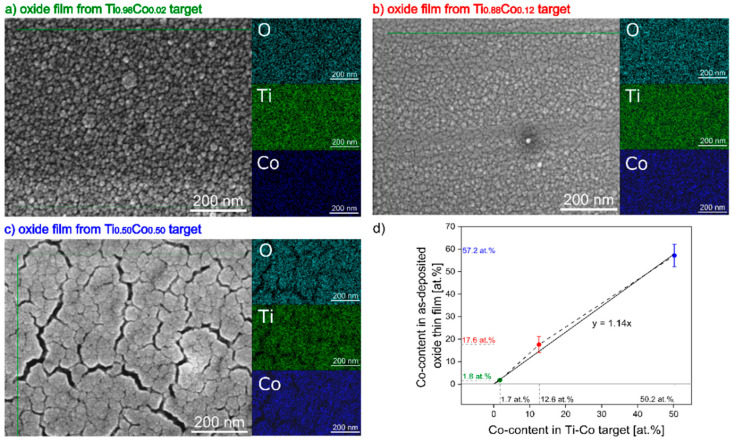
SEM images of surface topography with EDS maps of elemental distribution for oxide (Ti,Co)Ox thin films as deposited from: (**a**) Ti_0.98_Co_0.02_, (**b**) Ti_0.88_Co_0.12_, (**c**) Ti_0.50_Co_0.50_ targets; with (**d**) a diagram of the dependence between Co-content in the target and in the oxide film.

In Figure 3, the SEM images of surface topography with EDS maps of elemental distribution for oxide (Ti,Co)Ox thin films as deposited from Ti_0.98_Co_0.02_, Ti_0.88_Co_0.12_ and Ti_0.50_Co_0.50_ targets are shown. These results were used to draw up a diagram (Figure 3d) showing the relationship between the composition of the target and the oxide film. EDS maps of Ti, O and Co indicate a homogeneous distribution of these elements and a lack of agglomeration effects. The absence of areas with a clearly higher concentration of titanium or cobalt is important. This proves the good quality of sintered targets and the possibility of manufacturing coatings with a homogeneous composition based on them. Moreover, it was found that in the oxide films, there was more Co compared to its content in the target. The factor of 1.15 can be used to estimate the cobalt content of oxide coatings made from sintered Ti–Co targets. The 50 at.% amount of cobalt also causes stresses in the structure, which results in the presence of nano cracks in the microstructure of the (Ti,Co)Ox film (Figure 3c). Detailed results of EDS analysis are collected in Table 1.

The relationship between the composition of Ti–Co targets and the content of cobalt in as-deposited metallic and oxide films can also be analyzed based on the detailed data, which are collected in Table 1. As can be seen, there was relatively 15% ÷ 20% more cobalt in the films. These differences result from the sputtering yield of Co and Ti. In the case of oxide and metallic coatings deposited from the Ti_0.88_Co_0.12_ target, these differences are the greatest. However, it can be assumed that they result rather from the segregation of the titanium and cobalt atoms that were obtained with this target composition (see Figure 1b).

#### 3.1.4. Roughness of Metallic Ti–Co and Oxide (Ti,Co)Ox Thin Films

**Figure 4:**
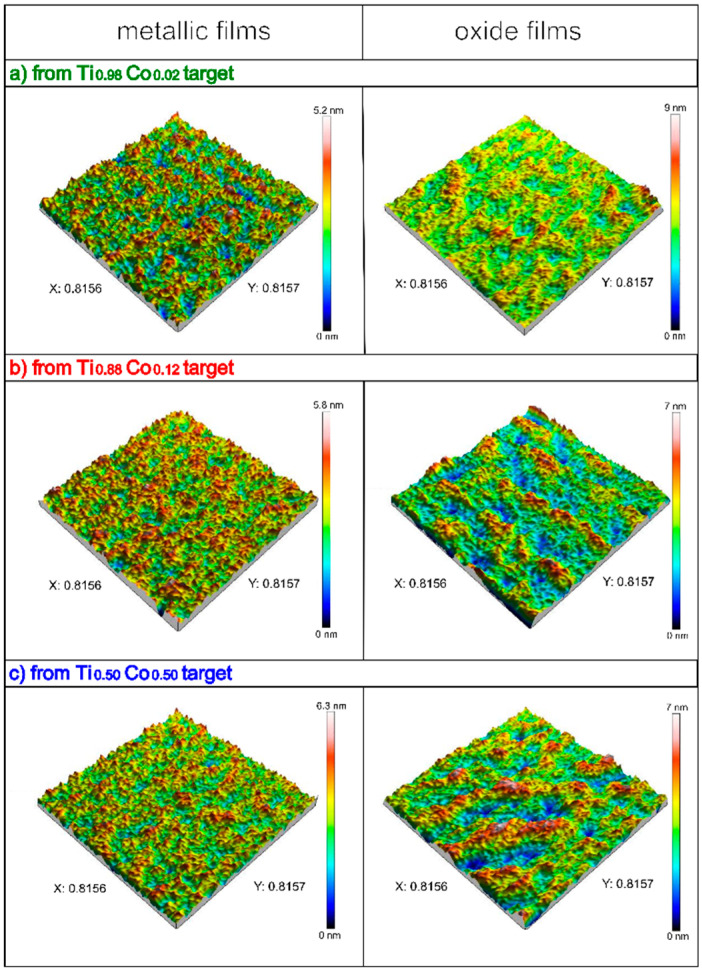
Three-dimensional profiles of metallic Ti–Co and oxide (Ti,Co)Ox thin films prepared by magnetron sputtering from different targets.

**Figure 5:**
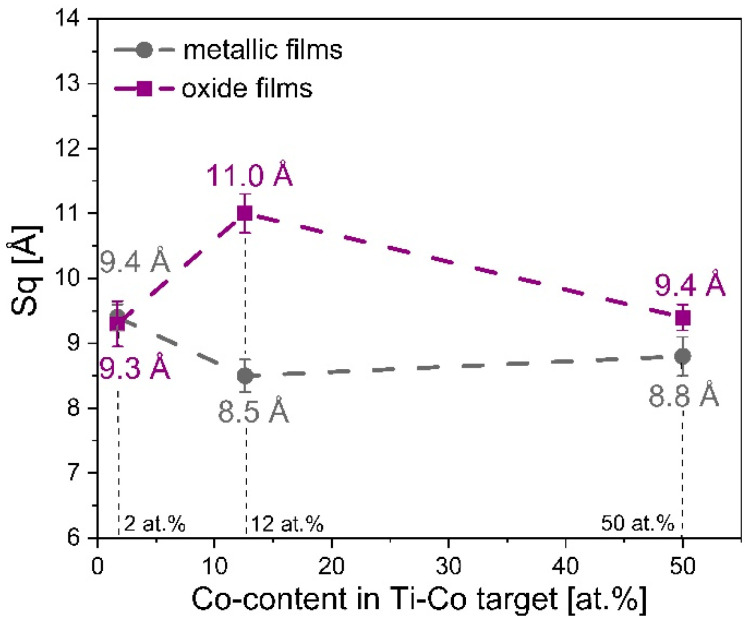
The influence of the Co-content in the Ti–Co target on the roughness of metallic and oxide thin films. Designation: Sq—root mean square roughness.

The influence of target composition on its sputtering rate under argon—as an inert atmosphere as well as in Ar:O_2_ gas mixture (with 30% of oxygen)—as an oxidizing atmosphere was also studied with the aid of an optical profilometer. Figure 4 shows the three-dimensional surface profiles of metallic Ti–Co and oxide (Ti,Co)Ox coatings. As it can be seen, all prepared films were smooth and homogenous. A detailed analysis, based on the Sq parameter (the root mean square height of the surface), showed that the roughness was at the level of 1 nm ± 0.2 nm (Figure 5). However, metallic films are characterized by almost the same Sq value (0.9 nm), which can also be concluded based on their 3D profiles (almost identical). In the case of oxide (Ti,Co)Ox films, the values of Sq parameter are also very similar, although slight differences in surface topography can be observed on the 3D profiles.

#### 3.1.5. Sputtering Rate of T-Co Targets for Metallic and Oxide Films

**Figure 6:**
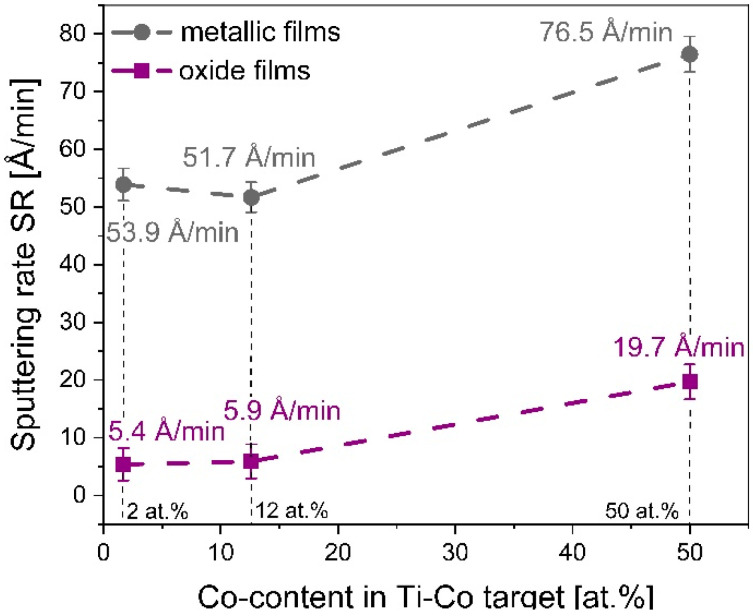
The influence of the Co-content in the Ti–Co target on the sputtering rate (SR) for metallic and oxide thin films.

Besides the roughness analysis, optical profilometry was also used for the determination of the film thickness. In the case of metallic Ti–Co films, the thickness was 324 nm, 310 nm and 460 nm (for 60 min of sputtering) for Ti_0.98_Co_0.02_, Ti_0.88_Co_0.12_ and Ti_0.50_Co_0.50_, respectively. The thickness of the oxide (Ti,Co)Ox films was 65 nm, 70 nm and 236 nm (for 120 min of sputtering), respectively. These results were in good agreement with SEM cross-section observations. Taking into account the deposition time of each coating and the construction of the substrate holder (drum with six sides), the sputtering rate was determined (Figure 6).

A common conclusion for all coatings is the fact that an increase in the amount of cobalt in the target causes an increase in its sputtering rate and thus the deposition rate of both metallic and oxide coatings. In the case of the Ti_0.98_Co_0.02_ and Ti_0.88_Co_0.12_ targets, the *SR* values are about 10-times higher in the argon itself than in the argon-oxygen gas mixture and are around 5 Å/min and 50 Å/min, respectively. For a higher cobalt content of the target, i.e., 50 at.%, the sputtering rate was approximately 20 Å/min and 75 Å/min in the Ar and Ar:O_2_ atmosphere, respectively. This proves that the use of an oxidizing atmosphere most likely causes the target poisoning effect and reduces its sputtering efficiency [28,29,30].

### 3.2. Structural, Optical and Electrical Characterization of (Ti,Co)Ox Thin Films

#### 3.2.1. Structure of Oxide (Ti,Co)Ox Coatings

**Figure 7:**
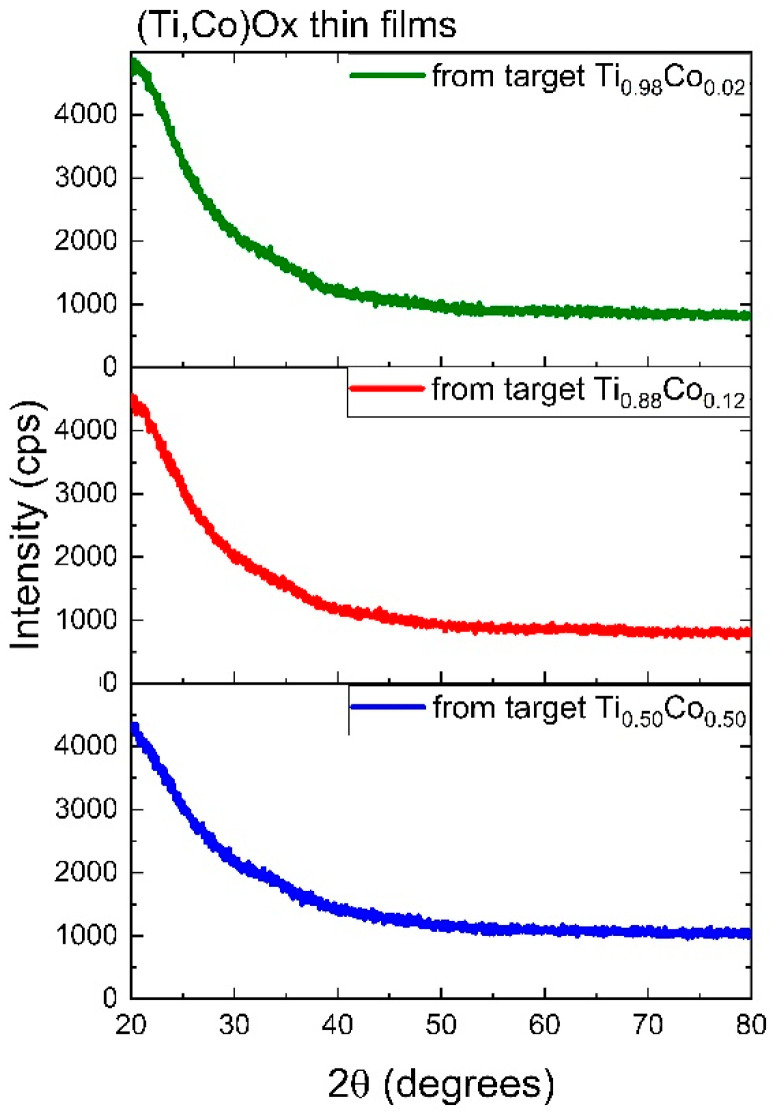
GIXRD patterns of oxide (Ti,Co)Ox thin films as deposited from different targets.

The influence of material composition (Co-content) on the structure of the as-deposited oxide coatings was examined by GIXRD. In Figure 7, the diffraction patterns of (Ti,Co)Ox films are shown. As it can be seen, their amorphous nature was identified. Only a background boost related to the amorphous SiO_2_ substrate can be observed in the 2θ range of ca. 20 ÷ 35. It should be emphasized that even for very thin coatings, their crystalline nature would be identified in grazing incidence measurements (GIXRD). In the case of pure titanium-based oxide coatings that were previously manufactured under the same conditions (described by the authors elsewhere [31,32]), a well-crystallized anatase structure (composed of crystallites with an average size less than 10 nm) was observed. This means that the addition of cobalt, both in small and large quantities, causes the amorphization of thin films and prevents crystallization. Similar results were obtained by Bohoroquez et al. [33] or Quiroz et al. [24], where Co-doped TiO_2_ films prepared by DC magnetron sputtering were completely amorphous. The presence of such crystalline phases as Co_3_O_4_, Ti_4_O_7_, CoTiO_3_ and CoTi_2_O_5_ usually requires additional annealing at high temperatures (above 450 °C). However, there are reports where the inclusion of such ions with lower valence states, as Co^2+^ and Co^3+^ increases the crystallization of TiO_2_ as well as the anatase to rutile transition [34]. The examined (Ti,Co)Ox thin films were sputtered in Ar:O_2_ (70:30) plasma. Such an atmosphere is rich in oxygen enough to obtain fully oxidized TiO_2_ films, while the formation of cobalt oxides (also well-crystallized) requires a higher portion of oxygen during the sputtering process [35]. Besides, low Co-content may also favor the growth of TiO_2_:Co, permitting the diluted formation [24,35]. In our opinion, cobalt ions block the process of crystallite nucleation and prevent further ordering that resulted in the amorphous nature of the obtained films.

#### 3.2.2. Microstructure and Morphology of Oxide (Ti,Co)Ox Coatings

**Figure 8:**
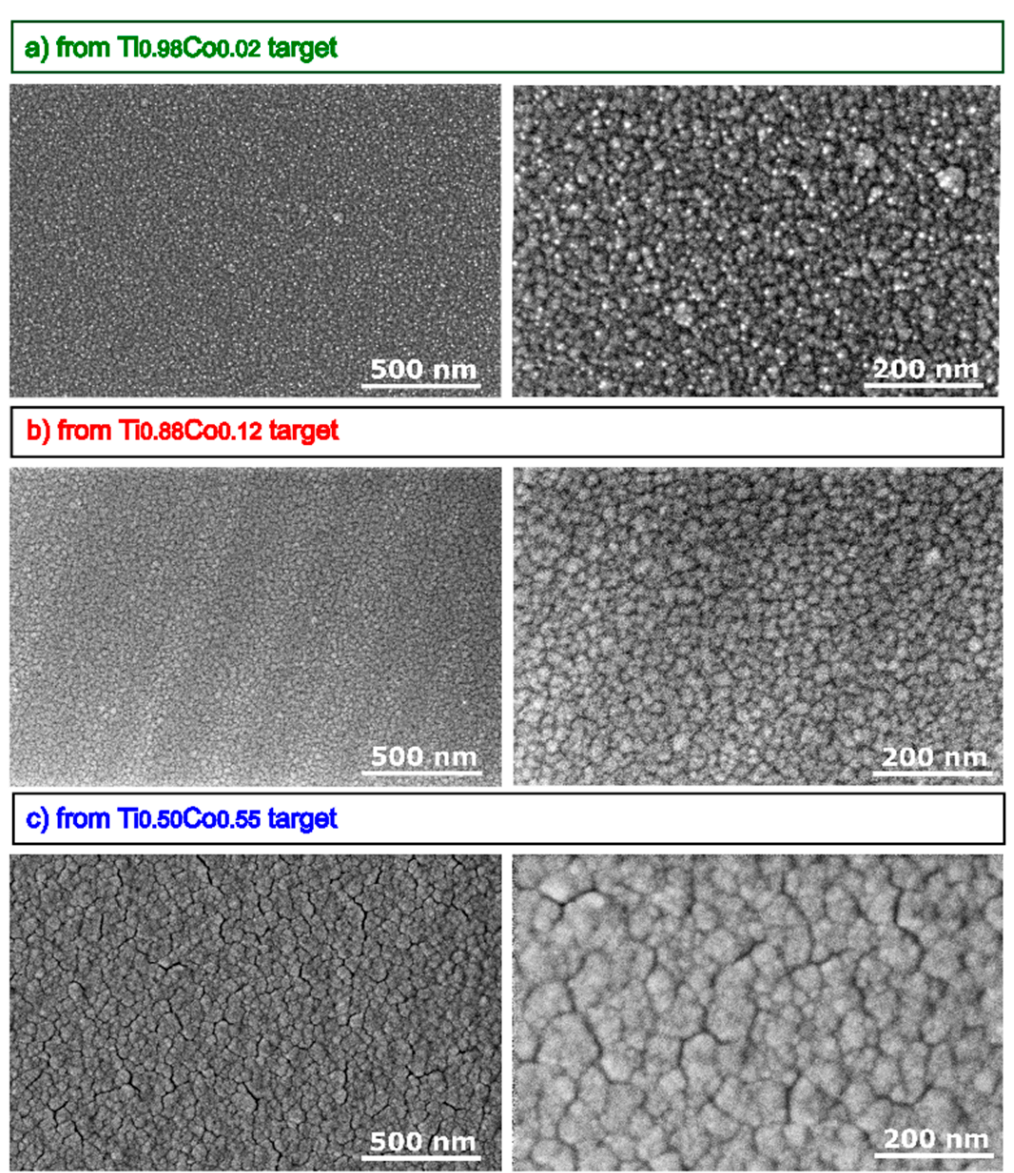
SEM images of surface of (Ti,Co)Ox thin films prepared from: (**a**) Ti_0.98_Co_0.02_, (**b**) Ti_0.88_Co_0.12_, (**c**) Ti_0.50_Co_0.50_ targets.

**Figure 9:**
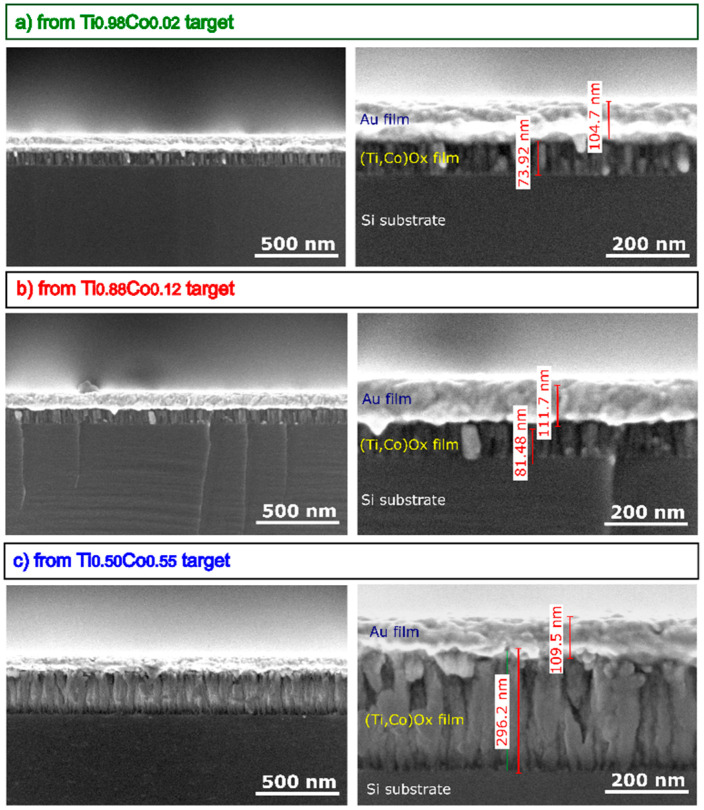
SEM images of cross-section of (Ti,Co)Ox thin films prepared from: (**a**) Ti_0.98_Co_0.02_, (**b**) Ti_0.88_Co_0.12_, (**c**) Ti_0.50_Co_0.50_ targets (with Au layer on top).

The influence of the cobalt content on the structural properties of the oxide coatings was also investigated with the aid of SEM. For cross-section imaging, a 100 nm thick Au thin film was evaporated on the top of TiO_2_:Co coatings to minimize the charging effect during SEM investigation and to obtain high-resolution images. Images of the surface topography (Figure 8), as well as cross-sections (Figure 9), revealed a densely packed columnar structure of all films. In particular, as the amount of cobalt in the films increases, the width of the columns increases (on average from 20 nm to 50 nm). A similar effect can be observed on the surface, which was built from the tops of densely arranged columns. An increase in their diameter is related to the increase in Co-content. Moreover, in the case of films sputtered from targets with 2 at.% and 12 at.% of Co, their microstructure is free of cracks or voids between columns, which can be seen in the case of the film obtained from the Ti_0.50_Co_0.50_ target. Based on SEM images, the deposition in the so-called “T zone” or “transition zone” can be identified, which is characterized by the presence of very small and elongated grains due to the superficial diffusion contribution to the species mobility among grains [36]. The characters of the nucleation process, associated with the substrate temperature and deposition method, indicate the random distribution of cobalt ions in the TiO_2_ matrix. Such conclusions were also reached in the work of Quiroz et al. [24]. Moreover, other works in the literature on TiO_2_:Co also indicate the effect of fine crystallinity of the grains caused by the addition of cobalt [33,37].

#### 3.2.3. Optical Properties of Oxide (Ti,Co)Ox Coatings

**Figure 10:**
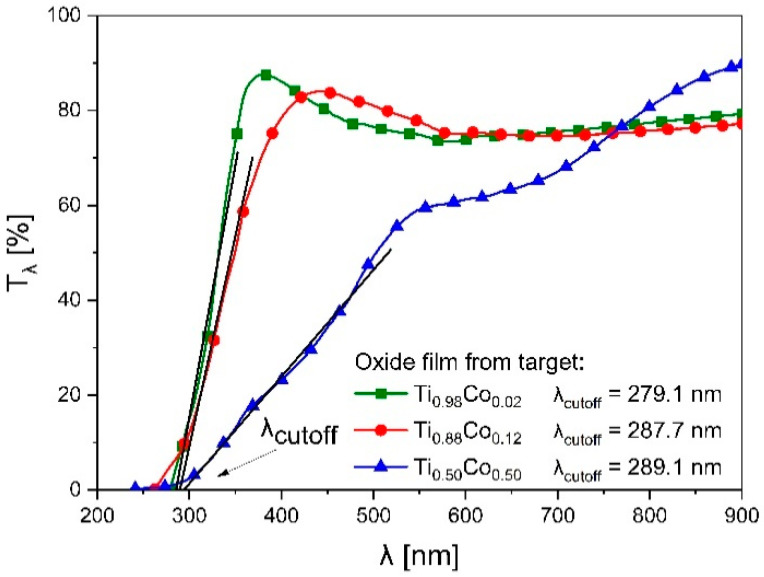
Transmission characteristics of (Ti,Co)Ox thin films prepared from different targets. Designations: T_λ_—light transmission coefficient, λ_cutoff_—position of the optical absorption edge.

The characterization of the optical properties of (Ti,Co)Ox coatings was performed on the basis of light transmission characteristics (Figure 10). It was found that all films exhibit high transparency. The value of T_λ=550 nm_ was ca. 75% for coatings deposited from Ti_0.98_Co_0.02_ and Ti_0.88_Co_0.12_ targets. However, 50 at.% of Co in the target resulted in the manufacturing of less transparent film, with transparency close to 60%. It is noteworthy that the oxide films based on titanium and cobalt are often characterized by a very low level of light transmission [33], and only their additional annealing at a temperature above 500 °C allows to obtain high transparency (usually in the range of 60% ÷ 70%).

**Figure 11:**
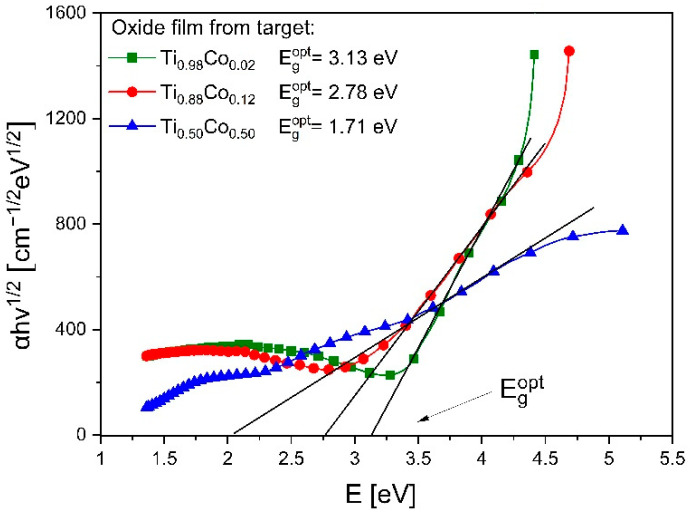
Tauc plots for (Ti,Co)Ox thin films prepared from different targets. Designations: E_g_^opt^—optical band gap.

**Figure 12:**
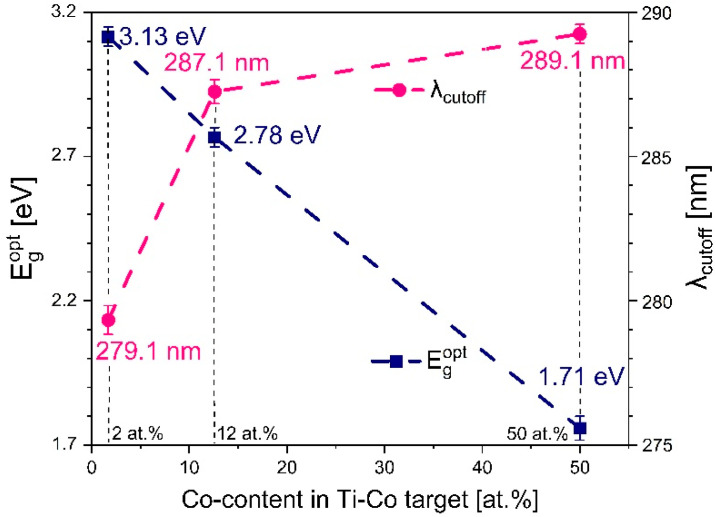
Influence of Co-content in the Ti–Co target on the optical band gap (E_g_^opt^) and position of the optical absorption edge (λ_cutoff_) of (Ti,Co)Ox thin films.

Based on the transmission characteristics, the Tauc plots (for indirect transitions) were determined, and the optical band gap (E_g_^opt^) was estimated (Figure 11). As it can be seen, the value of E_g_^opt^ was equal to 3.13 eV, 2.17 eV and 1.71 eV for the coatings as deposited from targets with 2 at.%, 12 at.% and 50 at.% of Co, respectively. This means that the greater the amount of cobalt in the (Ti,Co)Ox film, the lower the width of its optical band gap. Such effect was also noticed in other works [14,21]. In most cases, similar to the results described in this paper, an increase in the amount of cobalt causes a decrease in the E_g_^opt^ value. Our results of E_g_^opt^ were also presented as a function of the amount of cobalt in the target (Figure 12). They were compared with the values of the position of the optical absorption edge (λ_cutoff_). In this case, the effect of Co-content was also observed as a so-called “red shift” (towards longer wavelengths of light). Such effect was also reported in other works. For example, Subramanian et al. [18] or Musa et al. [20] in transparent TiO_2_:Co films also obtained a shift of λ_cutoff_ towards longer wavelengths due to a large amount of cobalt. It should be noted that the position of the optical absorption edge is mostly determined by the manufacturing technique and the process parameters. Therefore, the “blue shift” of the λ_cutoff_ can also be found [15].

#### 3.2.4. Electrical Properties of Oxide (Ti,Co)Ox Coatings

**Figure 13:**
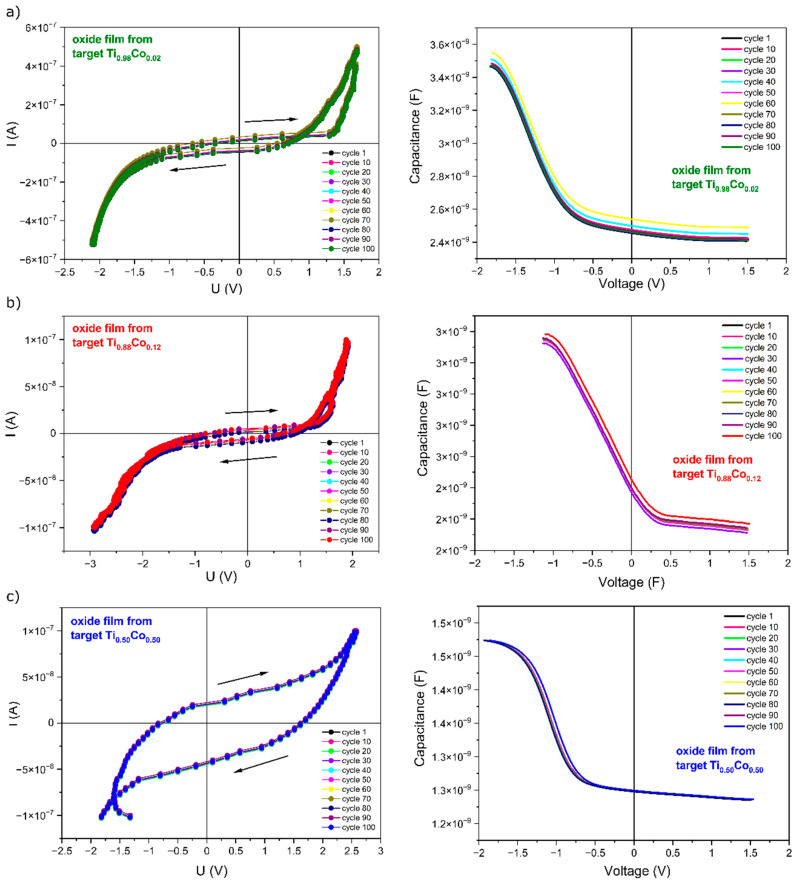
Current to voltage (I–V) and capacitance to voltage (C–V) characteristics of prepared Au/(Ti,Co)Ox/Si structures with different cobalt concentration—prepared from: (**a**) Ti_0.98_Co_0.02_, (**b**) Ti_0.88_Co_0.12_, (**c**) Ti_0.50_Co_0.50_ targets.

An investigation of electrical properties was performed for the prepared (Ti,Co)Ox thin films deposited on (p-type) silicon substrates. Additionally, a circular gold pad with 3 mm in diameter was deposited on the surface of the thin films to allow electrical measurements (Au/(TiCo)Ox/Si). That makes possible the discussion on the perspective application of prepared thin films in microelectronics. In Figure 13, the I–V characteristics measured in the transverse configuration are presented. I–V characteristics were presented in a conventional manner; the positive voltage/current values are due to the forward bias condition of the prepared structure, whereas negative values are due to the reverse bias condition.

During the measurements, the current was forced from negative to positive values and then back from the applied positive current values to the negative ones while the voltage drop was sensed using the source-measure unit (current-driven measurement). Sweeping the current allowed to observe the hysteresis loops, and as one can see in Figure 13a–c, but the shape of each of the measured curves is distinct. In the case of the structures with (Ti,Co)Ox thin films containing lower Co concentration, a unipolar memristive-like effect can be observed, which is manifested by the memory loop with forward bias polarization (Figure 13a) [38,39]. The occurrence of such effects has not been reported so far in the case of TiO_2_ coatings with the addition of Co. Usually, for these types of thin-film materials, a linear relationship between current and voltage is usually observed [15,20]. This means that materials based on titanium and cobalt oxides may be prospective for electronics, not only because of their spintronic application but also because of their memristive-like behavior. As it can be seen in Figure 13a, increasing the current during the measurement did not result in the change of shape of the measured characteristics until ca. 1.25 V. Above that voltage, the current suddenly increased in a relatively narrow voltage range (1.25 V ÷ 1.5 V). Changing the direction of the current flowing through the examined structure resulted in the formation of the hysteresis loop, indicating the occurrence of a memory effect. On the other hand, under the reverse bias condition, no hysteresis effect was observed, indicating the unipolar behavior of the observed phenomenon. A similar effect can be observed for the structure with (Ti,Co)Ox thin film containing about 17.6 at.% of cobalt (Figure 13b), but the memory effect, in this case, is much less pronounced (much narrower hysteresis loop). Quite unexpected results of the I–V measurement can be observed in Figure 13c. Measurements performed in similar voltage/current conditions, in this case, resulted in a visible very wide nonpinched hysteresis loop. We believe that this result was connected with a much thicker (Ti,Co)Ox thin film containing 57.2 at.% of Co, which resulted in a longer distance for electrical charge carriers that were injected from/to the metal electrode through the entire cross-section of the thin film. In this case, the observed wide hysteresis loop results rather from the polarisation and relaxation processes that occur in the thin oxide film and “charging” the structure upon switching the direction of the current flowing through it.

**Figure 14:**
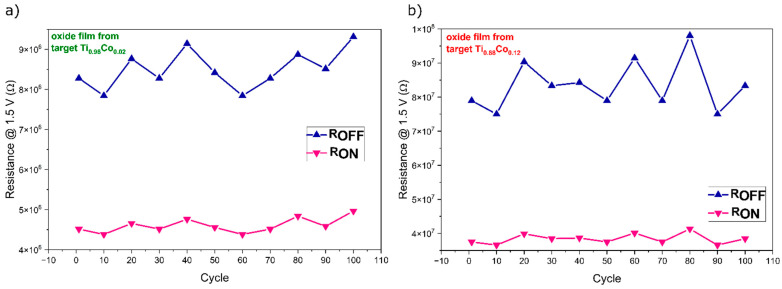
Retention plots of prepared Au/(Ti,Co)Ox/Si structures with different cobalt concentration—prepared from: (**a**) Ti_0.98_Co_0.02_ and (**b**) Ti_0.88_Co_0.12_ targets.

The capacitance-to-voltage characteristics were measured by sweeping the voltage bias from −2.0 V to 2.0 V using a 100 kHz sinusoidal measuring signal. The measurement was performed in a parallel electrical equivalent circuit. All three measured C–V curves showed a typical shape as for metal-oxide-semiconductor structures. The applied voltage sweeps the switching structure from accumulation to depletion state. Negative gate voltage at a forward bias condition causes the majority charge carriers (holes) in Si substrate to accumulate near the Si-oxide interface. Holes are injected from the Si substrate and are trapped in the oxide layer that results in a visible shift of the C–V curve toward negative voltages. For positive gate voltages, the Si-oxide interface is depleted, and in this state, the electrons from the inversion layer are injected into the oxide. Measured capacitance (c.a. 1.5 nF) in the accumulation state (oxide capacitance) was about two times lower for the structure with the highest Co-concentration (Figure 13c) than for the two remaining devices with much lower Co-concentration (c.a. 2.9 nF and 3.5 nF). This observation can be understood taking into account that the thickness of the thin film with the highest Co-concentration was more than two times higher. Measurements of current–voltage and capacitance–voltage characteristics performed for 100 equally repeated cycles testify to the high stability of the prepared thin-film structures. Additionally, retention plots for samples with switching behavior were constructed and presented in Figure 14. As one can see, in both cases, the maximum difference between R_ON_ and R_OFF_ resistance is quite similar.

## 4. Conclusions

The obtained results showed that the magnetron sputtering method is successfully suitable for the repeatable manufacturing of metallic and oxide thin-film coatings based on titanium and cobalt. In the case of the above-mentioned films, the deposition processes were carried out from Ti–Co targets with 2 at.%, 12 at.% and 50 at.% of cobalt. The relation between target composition and the Co-content in the metallic and oxide films was defined. There was relatively 15–20% more cobalt in the films than in the target. The deposition rate under neutral conditions (in Ar plasma) was much higher compared to oxidizing Ar:O_2_ plasma. GIXRD analysis of the structural properties revealed the amorphous nature of (Ti,Co)Ox coatings, regardless of the cobalt content in the coating. The fine-grained, homogenous microstructure was identified by SEM images, where cracks and voids were identified only for films with high Co-content. Optical studies have shown that as-deposited oxide films were well transparent (60% ÷ 80%), and the Co-content had a significant impact on the transparency level decrease as well as on the “red shift” of the optical absorption edge and the decrease in E_g_^opt^ (from 3.13 eV to 1.71 eV). Electrical studies revealed that in (Ti,Co)Ox thin films (with low cobalt content) deposited on Si substrate, a unipolar memristive-like effect can be observed. The occurrence of such effects has not been reported so far in the case of TiO_2_ coatings with the addition of Co.

## Figures and Tables

**Table 1:** Material composition of thin-film coatings (metallic and oxide) based on Ti and Co and targets used for their deposition by magnetron sputtering.

Target	Co-Content [at.%]		
	In the Target	In the Film	
		Metallic (Ti–Co)	Oxide (Ti,Co)Ox
Ti_0.98_Co_0.02_	1.7	3.5	1.8
Ti_0.88_Co_0.12_	12.6	19.4	17.6
Ti_0.50_Co_0.50_	50.2	60.7	57.2

## Data Availability

The data presented in this study are available in this article.
